# Modeling Parkinson's Disease Using Induced Pluripotent Stem Cells

**DOI:** 10.1155/2020/1061470

**Published:** 2020-03-12

**Authors:** Xinchao Hu, Chengyuan Mao, Liyuan Fan, Haiyang Luo, Zhengwei Hu, Shuo Zhang, Zhihua Yang, Huimin Zheng, Huifang Sun, Yu Fan, Jing Yang, Changhe Shi, Yuming Xu

**Affiliations:** ^1^Department of Neurology, The First Affiliated Hospital of Zhengzhou University, Zhengzhou University, Zhengzhou, 450000 Henan, China; ^2^Academy of Medical Sciences, Zhengzhou University, Zhengzhou, 450000 Henan, China

## Abstract

Parkinson's disease (PD) is the second most common neurodegenerative disease. The molecular mechanisms of PD at the cellular level involve oxidative stress, mitochondrial dysfunction, autophagy, axonal transport, and neuroinflammation. Induced pluripotent stem cells (iPSCs) with patient-specific genetic background are capable of directed differentiation into dopaminergic neurons. Cell models based on iPSCs are powerful tools for studying the molecular mechanisms of PD. The iPSCs used for PD studies were mainly from patients carrying mutations in synuclein alpha (*SNCA*), leucine-rich repeat kinase 2 (*LRRK2*), PTEN-induced putative kinase 1 (*PINK1*), parkin RBR E3 ubiquitin protein ligase (*PARK2*), cytoplasmic protein sorting 35 (*VPS35*), and variants in glucosidase beta acid (*GBA*). In this review, we summarized the advances in molecular mechanisms of Parkinson's disease using iPSC models.

## 1. Introduction

Parkinson's disease (PD) is the second most common neurodegenerative disease, which is characterized by static tremors, rigidity, bradykinesia, and postural instability. Widespread neuronal loss occurs in PD patients' brain, especially the progressive degeneration of dopaminergic neurons in the substantia nigra compacta [1]. The surviving neurons present inclusion bodies (Lewis bodies) containing *α*-synuclein in the central and peripheral nervous systems [2]. Genetic factors contribute significantly to the complex pathogenesis of PD [3]. 10% patients with hereditary PD carry disease-causing mutations, while most patients with sporadic PD may carry single nucleotide polymorphisms [4]. Common PD-related mutant genes include the synuclein alpha (*SNCA*), leucine-rich repeat kinase 2 (*LRRK2*), PTEN-induced putative kinase 1 (*PINK1*), parkin RBR E3 ubiquitin protein ligase (*PARK2*), and cytoplasmic protein sorting 35 (*VPS35*). Among them, *SNCA*, *LRRK2*, and *VPS35* are associated with PD in autosomal dominant forms, and *PINK1* and *PARK2* are associated with PD in autosomal recessive forms. In addition, genome-wide association studies have found that plenty of variants in glucosidase beta acid (*GBA*) are risk factors for PD [5].

At the cellular level, the molecular mechanisms of PD involve oxidative stress, mitochondrial dysfunction, autophagy, axonal transport, and neuroinflammation [5]. Increased oxidative stress products can damage macromolecules and cause mitochondrial dysfunction, which subsequently triggers mitochondrial autophagy. These pathways converge in the accumulation and aggregation of alpha-synuclein, a marker of PD. And the PD-related mutant genes may play multiple roles in these pathways, which is complex and elusive.

The emergence of induced pluripotent stem cells (iPSCs) has greatly promoted the research process of PD molecular mechanism. IPSCs are cells that resemble embryonic stem cells by transferring OCT4, Sox2, Klf4, and c-Myc (Yamanaka factor) retroviruses to somatic cells [6, 7]. Reprogrammed iPSCs have multiple differentiation potentials and are capable of self-renewal, similar to embryonic stem cells. More importantly, iPSCs have a patient's complete genomic background, providing a platform to more directly investigate the impact of genetic mutations on disease occurrence. Park et al. were the first to successfully establish iPSC models from PD patients [8]. And Soldner et al. differentiated iPSCs into dopaminergic (DA) neurons for the first time [9]. Subsequently, more and more iPSC models were established and differentiated into neurons to simulate the phenotype of PD [10]. The CRISPR/Cas9 system, an RNA-based endonuclease, can add/delete or modify genomes in living cells. Based on iPSC models, the CRISPR-Cas9 system has been effectively used for many purposes, such as allele-specific genome-targeted knockout [11] and knock-in [12], regulation of endogenous gene expression [13, 14], and isogenic iPSC line correcting [15]. The establishment of iPSC models, CRISPR/Cas9 system, and directional differentiation into neurons jointly control pathogenic genes as a single variable and eliminate phenotypic differences caused by individual inheritance, providing a more direct understanding of the relationship between specific genes and PD.

In this review, we summarized the current work on iPSC models with mutations in *SNCA*, *LRRK2*, *PINK1/Park2*, *VPS35*, and *GBA*. And we described the potentials and challenges of the iPSC models and their future development prospects.

## 2. Synuclein Alpha (*SNCA*)

**Figure 1:**
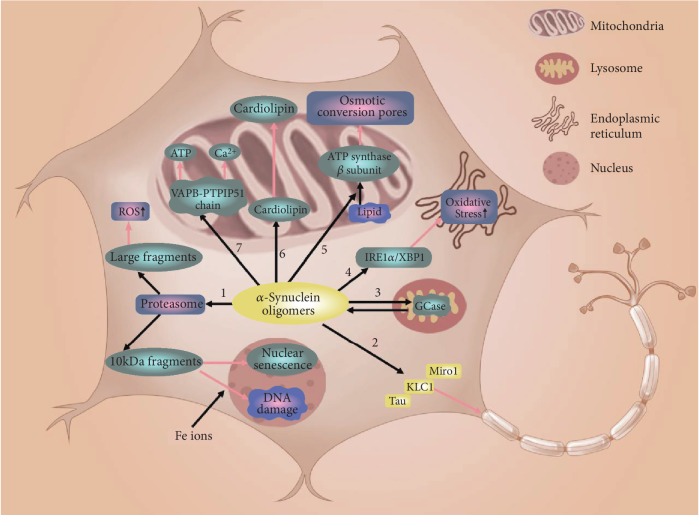
The role of *α*-synuclein in mDA neurons derived from SNCA triplication iPSCs: (1) hydrolyzed by proteases into large fragments and small fragments of 10 kDa. Large fragments increase ROS levels. Small fragments enter the nucleus and induce nuclear DNA damage and nuclear senescence. (2) Localizes to Miro1, KLC1, and Tau to impair mitochondrial axonal transport. (3) Interacts with GCase to promote lysosomal dysfunction. (4) Participates in IRE1/XBP1 axis to increase endoplasmic reticulum oxidative stress. (5) Promotes lipid and ATP*β* subunit binding to open mitochondrial pores. (6) Combines with cardiolipin to increase cardiolipin exposure at mitochondrial surface. (7) Interferes with VAPB-PTPIP51 chain to affect mitochondrial calcium and ATP balance.

*SNCA* was the first gene found in familial PD, which encodes *α*-synuclein (a core pathological marker of PD) [16]. The pathogenic *SNCA* reported mainly include point mutations (p.A53T, p.A30P, p.E64K, p.H50Q, p.G51D, and p.A53E), duplication, and triplication [17, 18]. Mutations or replication of *SNCA* makes *α*-synuclein conformational changes or dose increase, which leads to the occurrence of PD. The triplicated *SNCA* was first discovered in 2003 in an American family with PD [19]. Devine et al. were the first to establish iPSCs carrying *SNCA* triple replication and differentiated iPSCs into midbrain dopaminergic (mDA) neurons. These iPSC-derived dopaminergic neurons successfully mimicked the PD phenotype of *α*-synuclein accumulation, which were not detected in the skin fibroblasts from PD patients [20]. *SNCA*-related iPSC models are mainly derived from patients carrying *SNCA* triple replication, as this kind of model simulates the typical manifestations of PD. The role of *α*-synuclein in DA neurons derived from SNCA triplication iPSCs is depicted in Figure 1.

### 2.1. Oxidative Stress

The triplicated *SNCA* iPSC-derived DA neurons have a 2-fold increase in *α*-synuclein protein levels [20] and a 6-fold increase in mRNA levels [21]. The observed PD characterization of *SNCA* triplication iPSC-derived mDA neurons includes not only the accumulation of *α*-synuclein but also the intrinsic overexpression of oxidative stress markers and peroxide-induced oxidation [22]. Under environmental toxin or oxidative stress conditions, *SNCA* triplication iPSC-derived neural stem cells have higher vulnerability and increased oxidative stress sensitivity. Importantly, this phenotype can be reversed by knocking out endogenous *α*-synuclein [23]. Other studies have found that even small doses of *α*-synuclein are sufficient to induce large amounts of ROS. The resulting ROS, with free metal ion dependence, is induced by oligomers of *α*-synuclein rather than fibers [17]. Increased oxidative stress causes the imbalance of miRNAs in neurons [18], which is harmful to the nervous system [24]. Moreover, different oxidative stress signals produce different molecular effects in *SNCA* triple iPSC-derived DA neurons. Manganese results in a concentration- and time-dependent increase in intracellular ROS/nitrogen species, while rotenone causes an increase in intracellular lipid peroxidation (isoprostane) [25]. Remarkably, in *SNCA* triplication iPSC-derived cortical neurons, *α*-synuclein was found to induce endoplasmic reticulum stress by activating the unfolded protein response (UPR) of the IRE1*α*/XBP1 axis [26].

### 2.2. Nuclear Toxicity

Under physiological conditions, a small amount of *α*-synuclein is localized in the nuclei of neuronal cells [27]. When subjected to oxidative stress, extranuclear *α*-synuclein is cleaved by the proteasomes. Large fragments remain in the cytoplasm to increase stress-induced cell death, and small fragments in the C-terminal region translocate from the cytoplasm to the nucleus [28, 29]. *In vitro* and *in vivo* experiments have shown that *α*-synuclein can bind to chromatin [28] and activate the DNA damage response [30]. Another recent study showed that misfolded *α*-synuclein breaks the genomic DNA strand by opening a DNA nick. This DNA damage can be synergistic with Fe ions, promoting the death of *SNCA* triplication iPSC-derived neural progenitor cells [31]. In addition, *α*-synuclein may induce neurotoxicity by accelerating the cell cycle [32]. One study used a “seminatural” approach that prolongs culture time to induce senescence. Neurons from patients with *SNCA* triplication iPSCs developed earlier and faster nuclear senescence phenotypes, including nuclear folding as well as increased nuclear markers hp1*γ* and h3k9me3 [31]. Therefore, *α*-synuclein may mediate nuclear toxicity by impairing genomic integrity and accelerating senescence in *SNCA* triplication iPSC-derived neuronal nuclei.

### 2.3. Mitochondrial Toxicity

A high-throughput analysis showed that *SNCA* triplication iPSC-induced DA neurons harbored mitochondrial morphological changes and a decrease in mitochondrial membrane potential [33]. A transcriptomic analysis of purified *SNCA* triplication iPSC-derived DA neurons revealed perturbation of gene expression associated with mitochondrial function. This is consistent with the observed mitochondrial damage phenotype [34]. Animal and *in vitro* experiments showed that pathogenic *β*-sheet-rich *α*-synuclein oligomers are preferentially localized to mitochondria than wild-type *α*-synuclein, and accumulated *α*-synuclein deposits mediate mitochondrial dysfunction [35]. In *SNCA* triplication iPSC-derived DA neurons, *α*-synuclein (1) induces ATP synthase *β* subunit and mitochondrial lipid binding, opening osmotic conversion pores [36]; (2) binds to the endoplasmic reticulum-mitochondrial binding protein VAPB, disrupting the VAPB-PTPIP51 chain to relax the endoplasmic reticulum-mitochondrial association, Ca^2+^ homeostasis, and mitochondrial ATP production [37]; and (3) binds to the exposed cardiolipin on the mitochondrial outer membrane and increases the exposure time of the cardiolipin. Prolonged exposure of cardiolipin promotes refolding of the *α*-synthetic fibers and initiates recruitment of LC3 to mitochondria and mitochondrial autophagy [38]. Overexpression of ATP-dependent CLP protease reduces *α*-synuclein-induced mitochondrial oxidative stress, inhibits *α*-synuclein s129 phosphorylation accumulation, and promotes neuronal morphology by increasing the restoration of superoxide dismutase-2 levels [39].

### 2.4. Lysosomal Dysfunction

Aggregated *α*-synuclein enhances autophagy activity to meet the needs of its degradation [40], while excessive *α*-synuclein can mediate the pathological manifestations of lysosomes [41]. Glucocerebrosidase and *α*-synuclein form a two-way pathogenic loop in synucleinopathy [42]. A study showed that in *SNCA* triplication iPSC-derived mDA neurons, accumulated *α*-synuclein disrupts RAB1a-mediated hydrolase transport and reduces lysosomal function through an abnormal association with the cis-Golgi-binding factor GM130. Overexpression of RAB1a restores the Golgi structure, improves hydrolase transport and activity, and reduces pathological *α*-synuclein in patient neurons [43]. Consistent with these findings, another study showed that in *SNCA* triplication iPSC-derived DA neurons, *α*-synuclein is reduced by a noninhibitory small molecule of *β*-glucocerebrosidase (GCase), which is sufficient to reverse the downstream cytopathies, including hydrolase maturation and perturbation of lysosomal dysfunction [44]. In addition, *SNCA* triplication causes excess *α*-synuclein to impair phagocytosis in iPSC-derived macrophages. And iPSC-derived macrophages stop the degradation of *α*-synuclein by blocking lysosome and proteasome paths [45].

### 2.5. Axon Dysfunction

Axon transport relies on microtubules and motor proteins (kinesins and dynein), which is the basis for maintaining neuronal homeostasis [46]. Animal studies have shown that synucleinopathy begins at the synaptic terminals [47–49]. Mild overexpression of the mutant *α*-synuclein oligomers significantly reduces microtubule stability and impairs neurite network morphology [50]. A further study confirmed that the acidic C-terminal region of the toxic *α*-synuclein fibrils interacted with the basic central region of Tau, interfering with Tau-promoted microtubule assembly [51, 52]. In *SNCA* triplication iPSCs, oligomers of *α*-synuclein are relocated using the transport regulatory proteins Miro1, KLC1, and Tau, affecting mitochondrial anterograde axonal transport. Moreover, the presence of high levels of *α*-synuclein leads to decreased axonal density and structural synaptic degradation of iPSC-derived neurons [53].

## 3. Leucine-Rich Repeat Kinase 2 (LRRK2)

**Figure 2:**
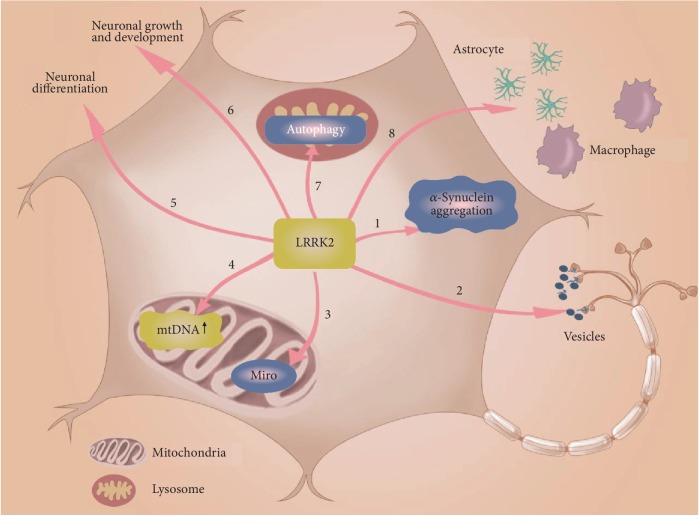
The role of mutant LRRK2 in iPSC-derived DA neurons. Mutant LRRK2 (1) promotes aggregation of *α*-synuclein, (2) interferes with the transport of axonal vesicles, (3) disrupts Miro-induced mitochondrial transport, (4) increases mitochondrial DNA levels, (5) interferes with neuronal differentiation, (6) interferes with neuronal growth and development, (7) increases lysosomal autophagy, and (8) regulates the immune function of macrophages and astrocytes.

LRRK2 is a protein with dual enzyme functions (GTPase and serine threonine kinase), which exists in the form of dimerization and binds to various organelle membranes to regulate the cytoskeleton [54]. LRRK2 participates in autophagy, immunity, and other physiological functions. The *LRRK2* G2019S mutation has the effect of enhancing LRRK2 kinase activity, and the first *LRRK2* iPSCs were established in 2012 [55]. Hereafter, *LRRK2* iPSC models carrying mutations G2385R [56], R1628P [57], N551K [58], and S1647T [59] were also established. The role of LRRK2 in iPSC-derived neurons is depicted in Figure 2.

### 3.1. Protein Homeostasis

LRRK2 interacts with *α*-synuclein. Increased *α*-synuclein level was found in *LRRK2* G2019S iPSC-derived neurons [60]. A study showed that LRRK2 could be able to modify *α*-synuclein pathology, and the presence of *LRRK2* G2019S enhanced the accumulation of endogenous *α*-synuclein in a time-dependent manner, accelerating neuronal degeneration, while *LRRK2* deletion reduced aggregation [61]. In human neurons derived from *LRRK2* G2019S iPSCs, *LRRK2* G2019S rapidly internalized recombinant human preformed-fibril, triggering the accumulation of endogenously expressed *α*-synuclein. This demonstrates that LRRK2 G2019S increases the formation of *α*-synuclein aggregates in patient neurons derived from iPSCs [61]. Furthermore, Daher showed that LRRK2 inhibitors can reduce neurodegeneration associated with abnormal *α*-synuclein accumulation [62].

### 3.2. Neuronal Differentiation

*LRRK2* mutations affect the ability of neurons to differentiate. Liu et al. found that iPSC-derived neural stem cells of *LRRK2* G2019S showed a passage-dependent defect in clonal expansion and neuronal differentiation [55]. In another study conducted by Bahnassawy et al., *LRRK2* R1441C neural stem cells were found to have impaired neuronal differentiation phenotypes, and *LRRK2* R1441C-deficient neural stem cells differentiated faster than wild-type cells [63]. Borgs et al. also demonstrated that *LRRK2* G2019S iPSCs are inefficient in the process of differentiation into DA neurons [64]. Further research on the specific role LRRK2 plays in neuronal differentiation is needed.

### 3.3. Neuronal Growth and Development

LRRK2 plays a role in neurite elongation and dendritization. The iPSC-derived sensory neurons of the *LRRK2* G2019S showed shortened neurites, reduced neurite outgrowth, microtubule-rich axon aggregation, and altered calcium dynamics. Treatment with LRRK2 kinase inhibitors can rescue this phenotype [65]. Borgs et al. reported significant branching defects in *LRRK2* G2019S iPSC-derived DA neurons [64]. Qing et al. found that in the *LRRK2* G2019S iPSC-derived mDA neurons, the percentage of TH-positive neurons with a total axon length greater than 2,000 *μ*m decreased significantly and the average branch of DA neurons decreased [12]. In addition, Korecka et al. recently confirmed that *LRRK2* G2019S can cause neuronal calcium-dependent phenotypic dysplasia. The *LRRK2* G2019S iPSC-derived mDA neurons had lower baseline ER-Ca^2+^ levels, while Ca^2+^ influx increased and Ca^2+^ buffering capacity decreased after membrane depolarization. After inhibiting the action of ER-Ca^2+^-ATPase, the *LRRK2* G2019S iPSC-derived neurons showed a neurite collapse phenotype [66].

### 3.4. Mitochondrial Dysfunction

About 10% of dimerized LRRK2 proteins are localized to the mitochondria and interact with substances on the mitochondrial membrane. Mutations in *LRRK2* G2019S can cause aberrations of mitochondrial morphology and function, an increase in mitochondrial number and mitochondrial debris, a decrease in mitochondrial membrane potential. This mitochondrial defect was found in the *LRRK2* G2019S iPSC-derived neuroepithelial stem cells in Walter's study [67]. Pathogenic LRRK2 mutations can induce mitochondrial genome damage and mitochondrial transport-related PD pathogenesis [68]. In Sanders et al.'s study, iPSC-derived DA neurons carrying the *LRRK2* G2019S or R1441C mutation showed high mitochondrial DNA (mtDNA) levels in iPSCs neurons when compared to normal iPSC-derived neurons. However, no mtDNA damage was found in iPSC-derived DA neurons, which were repaired by zinc finger nucleases [68]. Subsequently, another study confirmed that the mutated LRRK2 impairs mtDNA in a kinase-dependent manner. Inhibition of LRRK2 kinase activity can block or reverse mtDNA damage [69]. Notably, LRRK2 affects mitochondrial transport and impairs mitochondrial clearance. Under normal physiological conditions, LRRK2 forms a complex with the external mitochondrial membrane protein Miro. It promotes Miro removal and links PINK1 and parkin to Miro. Pathogenic *LRRK2* G2019S disrupts this pathway, arresting the movement of damaged mitochondria along the cytoskeleton and delaying mitochondrial autophagy [70]. Another study also confirmed mitochondrial distribution and trafficking abnormalities in LRRK2 mutant neurons, accompanied by significantly low endogenous NAD+ levels and decreased protein lysine deacetylase activity, leading to bioenergy defects [71].

### 3.5. Synaptic Vesicle Transport

The serine/threonine kinase activity of LRRK2 is important in the endocytosis of synaptic vesicles. *LRRK2* G2019S selectively impairs the endocytosis of synaptic vesicles in iPSC-derived ventral midbrain neurons (including DA neurons). Inhibition of LRRK2 kinase activity can rescue slow endocytosis. Through transcriptomics and proteomics analyses, Connor-Robson et al. found that *LRRK2* G2019S iPSC-derived DA neurons had a high degree of dysregulation of the inner circulation pathway [72]. The results revealed that a variety of key endocytic proteins were downregulated in cultures of *LRRK2* R1441C iPSC-derived DA neurons, such as endothelial cytokines I-III, dynamin-1, and various Rab proteins. Their study confirmed that clathrin-mediated endocytosis was disrupted [73]. Recently, Nguyen and Krainc reported that LRRK2 interacted with auxilin to jointly damage clathrin-mediated endocytosis of synaptic vesicles. They found auxin, which is phosphorylated by LRRK2, interfered with clathrin, resulting in disruption of synaptic vesicle endocytosis and decreased synaptic vesicle density in *LRRK2* iPSC-derived DA neurons [74]. LRRK2-mediated impaired synaptic vesicle endocytosis contributes to the accumulation of oxidized dopamine, producing dopamine-mediated toxic effects in iPSC-derived DA neurons, such as reduced glucocerebrosidase activity.

### 3.6. Autophagy

Normally, LRRK2 is degraded by proteasome and lysosomal pathways. The chaperone-mediated autophagy (CMA) pathway promotes lysosomal degradation of LRRK2. *LRRK2* G2019S was found to be involved in increased accumulation and release of *α*-synuclein [75]. *LRRK2* G2019S iPSC-derived mDA neurons showed higher levels of LC3 II than normal control cell lines, which represented the basal level of autophagy. This is possibly due to abnormal autophagosome clearance. Surprisingly, the phenotype of abnormal autophagy and neuronal damage in *LRRK2* G2019S iPSC-derived DA neurons can be rescued by the fission dynamin-related protein 1 (DRP1) peptide inhibitor p110 [76]. This suggests that mitochondrial hypermutation is involved in autophagy-associated PD mechanisms. In addition, leucine-tRNA synthetase (LRS) ligates leucine to tRNA Leu and activates rapamycin complex 1 (mTORC1). Ho et al. demonstrated that downregulation of LRS can enhance autophagy. LRRK2 phosphorylated LRS levels in the DA neurons of *LRRK2* G2019S, and LRS phosphorylation impaired autophagy through protein folding errors and endoplasmic reticulum stress mediated by LRS editing defects [77].

### 3.7. Neuroimmune Inflammation

In recent years, LRRK2 was found to be involved in the immune pathway of PD in both the central and peripheral systems, including innate immunity and acquired immunity [78]. LRRK2 is highly expressed in immune cells such as macrophages and microglia. Lopez de Maturana et al. found that *LRRK2* mutations affect *α*-synuclein regulation and impair NF-*κ*B classical signaling. LRRK2 silencing reduced *α*-synuclein levels in mutant neurons and NF-*κ*B dysregulation in mutant neurons. Moreover, NF-*κ*B dysregulation was found in mutant neurons [79]. In addition, Booth et al. found that matrix metalloproteinase 2 (mmp2) and transforming growth factor *β*1 (TGF*β*1) were downregulated in the cytoplasm of *LRRK2* G2019S iPSC-derived astrocytes, suggesting that *LRRK2* G2019S mutation may interfere with astrocytes [80]. Furthermore, *LRRK2* mutation resulted in accelerated production of *LRRK2* iPSC-derived monocytes and a decrease in noncanonical CD14^+^CD16^+^ monocyte subsets. The migration ability of these monocytes was found to be impaired. These results indicate that LRRK2 also plays a key role in hematopoiesis, supporting the pathogenic role of immunity in PD [81].

## 4. PTEN-Induced Kinase 1 (*PINK1*) and Parkin RBR E3 Ubiquitin Protein Ligase (*PARK2*)

**Figure 3:**
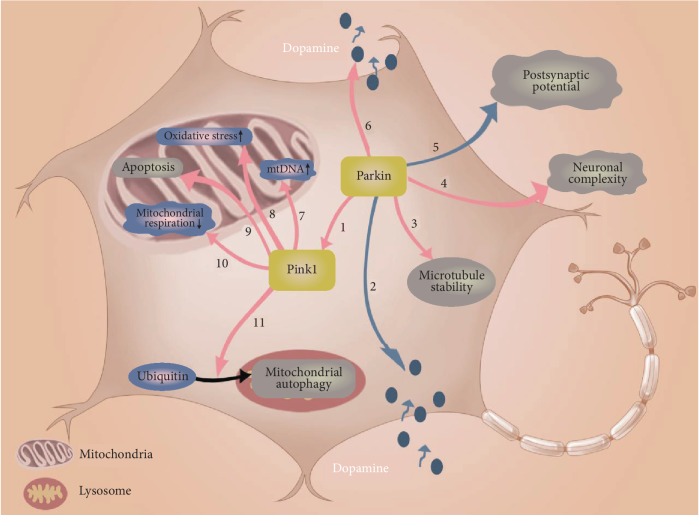
The roles of the mutant PINK1 and parkin in iPSC-derived DA neurons. Mutant PARK2 (1) impairs recruitment to PINK1, (2) reduces dopamine vesicle endocytosis, (3) disrupts microtubule stability, (4) reduces neuron complexity, (5) increases neuronal spontaneous excitatory postsynaptic currents, and (6) increases dopamine release. Mutant PINK1 (7) elevates mitochondrial DNA levels, (8) increases mitochondrial oxidative stress, (9) increases autophagy, (10) decreases mitochondrial respiration, and (11) interferes with ubiquitin-mediated mitochondrial autophagy and lysosomal autophagy.

PTEN-induced kinase 1 (*PINK1*) is a mitochondrial serine/threonine-protein kinase encoded by the *PINK1* gene. It is involved in the regulation of mitochondrial degradation and protects cells from stress. Parkin, produced by the *PARK2* gene, is involved in the maintenance of mitochondrial function and integrity. The role of PINK1 and parkin in iPSC-derived neurons is depicted in Figure 3.

### 4.1. Oxidative Stress

Initial studies have shown that PINK1 deficiency caused embryonic stem cell-derived dopaminergic neurons to exhibit significant oxidative stress characteristics, as these neurons died through the mitochondrial apoptotic pathway [82]. This phenotype was observed in the *PINK1* iPSC-derived neural cell population. Imaizumi et al. conducted a study of *PARK2* iPSC-derived neuron, observing the similar phenotypes with increased levels of oxidative stress. In addition, the Nrf2 pathway was activated in *PARK2* iPSC-derived neurons [83]. Another study showed a higher susceptibility to rotenone-induced mitochondrial stress in *PARK2* iPSC-derived DA neurons. This phenotype can be prevented by T-type calcium channel inhibition or antagonists. These studies have demonstrated the induction of oxidative stress in neurons by PINK1 and parkin [84].

### 4.2. Mitochondrial Dysfunction

IPSC-derived mDA neurons carrying *PINK1* and *PARK2* mutations showed PD pathology of mitochondrial dysfunction [85]. Cooper et al. observed elevated ROS, decreased mitochondrial respiration, proton leakage, and impaired mitochondrial movement in *PINK1* iPSC-derived neurons [86]. Seibler et al. found that in the presence of mutant *PINK1*, the mtDNA copy number increased and upregulation of PGC-1*α* in iPSC-derived DA neurons occurred, implying that PINK1 impaired mitochondrial function due to loss of function [87]. In Vos et al.'s study, the fatty acid synthase (Fasn) activity of *PINK1* iPSC-derived DA neurons decreased, resulting in decreased palmitate levels and increased cardiolipin (CL) levels. Importantly, increased cardiolipin can promote electron transfer between ubiquinone and complex I to rescue PINK1 deficiency [88].

Parkin also plays an important role in the mitochondria. *PARK2* iPSC-derived mDA neurons also exhibited mitochondrial dysfunction, abnormal mitochondrial morphology, decreased mitochondrial volume fraction, and impaired mitochondrial homeostasis. But these phenotypes were not observed in dermal fibroblasts and iPSCs [89]. This neuron-specific mitochondrial-damaged phenotype is consistent with that of previous studies [83]. Recent studies showed that parkin interacted with Stomatin-like protein 2 (SLP-2), which binds to mitochondria and functions in the assembly of the respiratory chain protein. Loss of parkin results in decreased complex I activity and increased mitochondrial fragmentation, whereas the overexpression of SLP-2 could rescue these phenotypes [90, 91]. It is worth noting that the *PARK2* mutation was found to affect the cellular energy metabolism rhythm. A recent study performed by Pacelli et al. has shown that iPSCs carrying the *PARK2* mutation and its differentiated neural stem cells were observed to be severely damped in the bioenergy oscillation mode [92].

### 4.3. Mitochondrial Autophagy

PINK1 initiates ubiquitin-mediated mitochondrial autophagy via parkin [93]. When the mitochondrial membrane potential is lost, PINK1 is degraded by the proteasome and accumulates on the damaged mitochondria while parkin is transported to the mitochondria in a PINK1-dependent manner, ubiquitinating the mitochondrial outer membrane protein (more of a large molecular weight protein) [94, 95]. Damaged mitochondria are labeled with polyubiquitin phosphorylation and cleared by mitochondrial autophagy to protect the neurons. A study showed that in *PINK1* iPSCs, both endogenous parkin and overexpressing parkin were insufficient to induce mitochondrial autophagy following the loss of mitochondrial membrane potential [94]. Another study found that in *PINK1* iPSC-derived DA neurons, mitochondrial recruitment was impaired under stress conditions, even overexpressing parkin. But the expression of wild-type PINK1 can rescue parkin-localized impaired mitochondrial dysfunction [87]. These two studies showed the important role of PINK1 in mitochondrial autophagy. Moreover, Oh et al. found that the S-nitrosylation of the Cys568 site of PINK1 downregulates its kinase activity, and S-nitrosylated PINK1 reduces parkin translocation to the mitochondrial membrane, disrupting iPSC-derived neuronal mitochondrial autophagy [96]. In addition, mitochondrial autophagy was also observed to be impaired in the iPSC-derived DA neurons of the *PARK2* mutation [97]. This signifies the importance of PINK1 and parkin in the mitochondrial autophagy pathway.

### 4.4. Dopamine Regulation

Another important function of parkin is to regulate dopamine in neurons. High levels of dopamine in the cytoplasm can lead to an increase in metabolites toxic to neurons, such as 6-hydroxydopamine [78]. *PARK2* iPSC-derived DA neurons showed decreased dopamine uptake and increased spontaneous dopamine release. So, parkin was presumed to control dopamine utilization in human mDA neurons by increasing the accuracy of dopamine neurotransmission and inhibiting dopamine oxidation [98]. A recent study has found that activation of dopamine D1 receptors in *PARK2* iPSC-derived midbrain neurons causes large rhythmic outbreaks of spontaneous excitatory postsynaptic currents (EPSCs) [99]. Importantly, Zhong et al. found that parkin's overexpression, but not its PD-causing mutant, abolished the oscillatory activity of the patient's neurons. These results indicate that *PARK2* mutations significantly enhance the regulation of abnormal dopaminergic regulation of presynaptic glutamate transmission in midbrain neurons [99].

### 4.5. Microtubule System

Microtubules transport the organelles necessary for outgrowth under normal physiological conditions. Previous studies have demonstrated that parkin bonds to microtubules with high affinity [100, 101] and stabilizes microtubules against toxicity [102]. Ren et al. found that the *PARK2* iPSC DA neurons significantly reduced complexity [103]. They used the microtubule depolymerizing agent colchicine to mimic the role of *PARK2* mutations by reducing the length and complexity of the control neuronal neurites, while the microtubule-stabilizing drug paclitaxel mimics the role of parkin overexpression by enhancing the morphology of parkin-deficient neurons. These results indicated that parkin maintained the morphological complexity of human neurons by stabilizing the microtubules. Another study conducted by Cartelli et al. reported that parkin defects caused stable microtubule fragmentation and accelerated acetylation in *PARK2*-mutated iPSC neurons [104]. These studies confirmed that parkin plays a regulatory role in the microtubule system during neuronal aging.

## 5. VPS35 Retromer Complex Component (*VPS35*)

*VPS35* encodes vacuolar protein sorting 35, which is a core component of the reversal complex. VPS35 localizes to dendritic spines and is involved in the recycling of proteins from the endosomes/lysosomes to the trans-Golgi network as well as from the endosomes to the plasma membrane [105]. The first two independent studies identified *VPS35* c.1858G>A (p.Asp620Asn) in the hereditary PD family in Switzerland [106] and the Austrian PD family [107]. Subsequently, mutations such as c.1570C>T (p.Arg524Trp) and c.946C>T (p.Pro316Ser) were also reported. Munsie et al. first established a dopamine neuron model with iPSCs sourced from patients with *VPS35* p.D620N. They found this loss-of-function mutation altered the transport of the episome-dependent neurotransmitter receptor to the synapse. This disturbance of synaptic function may place chronic pathophysiological stress on the neuronal circuit [108]. Currently, there are few studies on the iPSC model based on *VPS35*. So it is necessary to further study the pathogenic mechanism of *VPS35* in the iPSC models.

## 6. Glucosidase Beta Acid (*GBA*)

**Figure 4:**
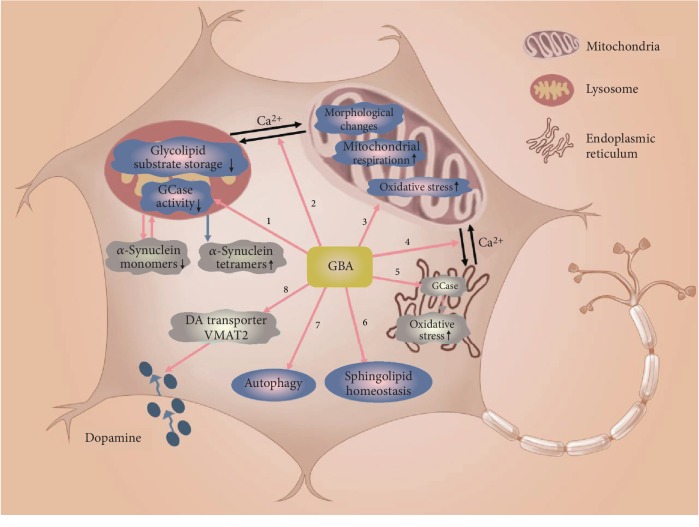
The role of mutated GBA in iPSC-derived DA neurons. Mutant GBA (1) reduces GCase activity in lysosome and increases glycolipid substrate storage leading to lysosomal dysfunction; (2) disrupts calcium balance between mitochondria and lysosomes; (3) changes mitochondrial morphology, reduces mitochondrial respiration, and increases oxidative stress in mitochondria; (4) disrupts calcium balance between endoplasmic reticulum and mitochondria; (5) destroys GCase in endoplasmic reticulum to increase oxidative stress in endoplasmic reticulum; (6) disrupts intracellular sphingolipid homeostasis; (7) increases autophagy; and (8) reduces DA transporter and VMAT2 levels to interfere with dopamine uptake. Lysosomal dysfunction reduces *α*-synuclein monomers and accumulates *α*-synuclein tetramers.

*GBA* encodes a hemolytic hydrolase *β*-glucocerebrosidase (GCase). GCase degrades glucosylceramide (GluCer) to glucose and ceramide in lysosomes. The *GBA* mutation is the strongest risk gene for PD [109]. Common *GBA* mutations are N370S and L444P. Woodard et al. first established *GBA* iPSC-derived DA cells from single-oval twins carrying with the *GBA* N370S mutation [110]. The role of GBA in iPSC-derived neurons is depicted in Figure 4.

### 6.1. Protein Homeostasis

Woodard et al. found that GBA enzyme activity was lower and *α*-synuclein levels were significantly elevated [110]. Another independent study demonstrated that in *GBA* iPSC-derived DA neurons, *GBA* mutations resulted in decreased glycosidase activity and storage of glycolipid substrates [111]. Correspondingly, *α*-synuclein aggregation occurs in iPSC-derived mDA neurons exposed to GCase inhibitors. Kim et al.'s study has shown that the lack of GCase reduces the aggregation of physiologically formed *α*-synuclein tetramers and increase the presence of *α*-synuclein monomers, leading to neurotoxicity. Importantly, overexpression of GCase reverses this process [112]. In addition, glucosyl sphingosine (GlcSph) and sphingosine (Sph), members of the lipid family of ceramides, potently promoted the accumulation of pathological *α*-synuclein in *GBA* iPSC-derived neurons [113]. A study has shown that mutated GBA reduces the function of GCase and increases the accumulation of *α*-synuclein, which may be possible through the autophagolysosomal pathway that disrupts *α*-synuclein [114]. The increased aggregation of *α*-synuclein feedback inhibits the activity of glucocerebrosidase, and this bidirectional circulation leads to the development of *GBA*-associated PD.

### 6.2. Pathological Mechanism of PD Mediated by GBA Mutation

In iPSC-derived DA neurons carrying the *GBA*-N370S mutation, *GBA* mutation disrupted the physiological structure of GCase in the endoplasmic reticulum, activated the unfolded protein response (UPR), and upregulated endoplasmic reticulum stress. In addition, the reduced activity of GCase impairs autophagy/lysosomal system function and expands the lysosomal compartment, making dopamine neurons susceptible to individual recognition. No increase in *α*-synuclein levels was observed in neurons of iPSC-derived DA neurons that were not mutated in *GBA*-N370S, but increased levels of extracellular *α*-synuclein release in culture [114]. Another study also reported that the *GBA* iPSC-derived mDA neurons were damaged by the autophagy system. It is worth noting that *GBA* mutant neurons also showed dysregulation of calcium homeostasis and increased susceptibility to calcium-induced stress responses [115]. Importantly, reduced levels of DA transporter and VMAT2 expression are shown in PD neurons, which may help reduce DA absorption in these cells [111]. In addition, GlcCer and GlcSph accumulation has been detected in *GBA*-KO iPSCs neuron mitochondria [116]. In *SNCA* iPSC-derived neurons, GlcCer levels and decreased ceramide levels were found to be elevated [43]. In conclusion, mutated *GBA* may induce neuronal PD phenotype through endoplasmic reticulum stress, autophagy/lysosomal dysfunction, and calcium homeostasis.

## 7. iPSC Models Confirm the Neuron-to-Neuron Transmission of *α*-Synuclein

*α*-Synuclein, a key factor triggering PD, multiplies between cells in a prion-like manner, whose protein aggregates bind heparan sulfate proteoglycans (HSPGs) on the cell surface to transmit pathologic processes [117]. The exogenous *α*-synuclein fibrils are assembled with heparan sulfate proteoglycan [118] as well as membrane proteins on the cell surface. These fibrils are taken up by intracellular endocytosis and participate in intracellular direct and retrograde transport [119]. Exogenous *α*-synuclein acts as a template to promote endogenous *α*-synuclein from a physiological *α* helix to an insoluble beta-fold conformation, aggregating protease K-resistant oligomeric fibrils. This pathogenic process exists not only between neuronal cells but also within the brain regions where the nervous systems are interconnected.

The theory of iPSC-derived human neuron models confirms the spread of *α*-synuclein between neurons. Yamasaki et al. demonstrated the propagating seed characteristics of *α*-synuclein insoluble monomers [120, 121]. Gribaudo et al. established a network of healthy human neurons in a cortical neuron network of microfluidic devices to find that *α*-synuclein multiplies between neurons in a dose- and structure-dependent manner, triggering PD-like pathology [122]. In addition, Surguchev et al. showed that extracellular *α*-synuclein interacts with cell membrane receptors such as cytoplasmic protein, lymphocyte activating gene 3, and Toll-like receptor 2. Cell signaling promotes *α*-synuclein to propagate between different cells [123]. The above results together indicate that in the neurons of patients with familial Parkinson's disease caused by genetic mutations, their *α*-synuclein pathology has sufficient seed characteristics to cause age-dependent human neuronal degeneration spread in brains.

## 8. The Potentials and Challenges of iPSC Technology

Considering the insertion of oncogenes c-Myc and Klf4 increases the risk of mutation and transformation into cancer cells, subsequent studies have made various improvements in reprogramming methods. Soldner et al. used Cre recombinase after reprogramming to remove viruses and successfully obtained factor-free iPSCs that are more closely related to human embryonic stem cells [9]. Other researches performed more effective reprogramming procedure using safer vectors, such as nonintegrated vectors [124–126], synthetically modified mRNAs [127–129], cell membrane permeable proteins [130, 131], and small molecule compounds [132–134]. These technologies maximize genomic integrity and reduce the risk of transformation to cancer.

Genome-wide association analysis, sequencing of whole exomes and transcriptomes, has revealed an increasing number of disease genomics and proteomics, which greatly facilitates the study of neurodegenerative diseases [135]. In single-gene diseases, iPSCs that faithfully mimic disease phenotypes validate newly proposed disease mechanisms [136] and screen for therapeutic factors [137]. In polygenic diseases, iPSC library can be used to analyze the effects of SNPs and drug response differences [138]. iPSC technology and gene editing systems obtaining and target-editing of individual genome are potential strategies with great personalized treatment.

Dopamine supplementation and surgery are the first line of clinical treatment of PD to slow the progression of the disease [1]. Transplanted fetal midbrain cells were initially used in an attempt to treat poor endogenous nerve repair in patients with PD [139–141]. However, this technique has unavoidable limitations such as the uneven production of embryonic tissue and the susceptibility to genomic DNA damage during processing [141]. IPSCs have the advantages of avoiding genomic damage, enabling patients to adapt to HLA, high uniformity of cell grafts, and high proportion of dopaminergic neurons, which is also an ideal cell replacement therapy [127, 142]. In addition, cell-sorting techniques have been developed to reduce posttransplantation cancer risk, which target against the cell marker CORIN [143], the central nervous system microvascular endothelial marker LRTM1 [144], and activated leukocyte adhesion molecule (ALCAM) antibodies [145]. These cell-sorting technologies maintain the quality of transplanted cells and improve the safety and effectiveness of cell replacement therapy. Multiple animal trials have shown that iPSC transplantation is successful and safe in treating neurological diseases [146–148], and human clinical trials of Parkinson's disease using iPSCs are ongoing and observed [149–151]. Therefore, the iPSC treatment is expected to become a promising method for PD patients. In the near future, the clinical results of using iPSCs to treat Parkinson's disease are worth looking forward to.

Since the nervous system is not a single neuron but a complex culture system, the use of relatively simple 2D neurons in the disk modeling has not been able to meet the further need to explore PD. Various methods of cultivating 3D neural organs have been explored, such as Spin*Ω*'s microrotating bioreactors to build brain organs (forebrain, midbrain, and hypothalamus) [152], the SFEBq method to produce the “cerebellum” [153], and the production of human cortical spheres [154]. Recently, the method of cultivating 3D organs using neural rosettes established by dual signals of SMAD and FGF has greatly enhanced the reproducibility of brain organoids [155]. These 3D models based on iPSC-derived neurons will help people to more stereoscopically understand the occurrence and progression of PD.

PD is age-dependent and often occurs as a patient age. While the iPSC derivative is young and its culture period is short. Studies have reported that the length of the telomerase of iPSCs greatly increased during the reprogramming process [156, 157]. In addition, reprogrammed stem cells also rearrange the mitochondrial network and a lower oxidative stress phenotype [158]. This reflects that the younger performance of iPSCs is different from the phenotype of aging cells. Research groups have been building iPSC models that express aging markers, such as progerin [159] and astrocytes [160]. Currently, it is a challenge to simulate age-growth neurons and to more closely integrate aging systems with PD.

## 9. Conclusions

IPSCs offer a new platform for modeling and studying PD. While improvements still need to be made in iPSC-based disease modeling, this technology offers an unprecedented ability to mimic disease *in vitro* with patient-specific disease-relevant cell types. Human iPSC technology provides a more predictive platform for preclinical studies and improves the success of clinical trials, with the potential to deepen our understanding of the pathogenesis of disease.

## References

[1] Poewe W., Seppi K., Tanner C. M. (2017). Parkinson disease. *Nature Reviews Disease Primers*.

[2] Kalia L. V., Lang A. E. (2015). Parkinson's disease. *The Lancet*.

[3] Trinh J., Farrer M. (2013). Advances in the genetics of Parkinson disease. *Nature Reviews Neurology*.

[4] Lill C. M. (2016). Genetics of Parkinson's disease. *Molecular and Cellular Probes*.

[5] Cobb M. M., Ravisankar A., Skibinski G., Finkbeiner S. (2018). iPS cells in the study of PD molecular pathogenesis. *Cell and Tissue Research*.

[6] Takahashi K., Yamanaka S. (2006). Induction of pluripotent stem cells from mouse embryonic and adult fibroblast cultures by defined factors. *Cell*.

[7] Takahashi K., Tanabe K., Ohnuki M. (2007). Induction of pluripotent stem cells from adult human fibroblasts by defined factors. *Cell*.

[8] Park I. H., Arora N., Huo H. (2008). Disease-specific induced pluripotent stem cells. *Cell*.

[9] Soldner F., Hockemeyer D., Beard C. (2009). Parkinson's Disease Patient-Derived Induced Pluripotent Stem Cells Free of Viral Reprogramming Factors. *Cell*.

[10] Bahmad H., Hadadeh O., Chamaa F. (2017). Modeling human neurological and neurodegenerative diseases: from induced pluripotent stem cells to neuronal differentiation and its applications in neurotrauma. *Frontiers in Molecular Neuroscience*.

[11] Smith C., Abalde-Atristain L., He C. (2015). Efficient and allele-specific genome editing of disease loci in human iPSCs. *Molecular Therapy*.

[12] Qing X., Walter J., Jarazo J., Arias-Fuenzalida J., Hillje A. L., Schwamborn J. C. (2017). CRISPR/Cas9 and piggyBac-mediated footprint-free LRRK2-G2019S knock-in reveals neuronal complexity phenotypes and *α*-Synuclein modulation in dopaminergic neurons. *Stem Cell Research*.

[13] Soldner F., Stelzer Y., Shivalila C. S. (2016). Parkinson-associated risk variant in distal enhancer of *α*-synuclein modulates target gene expression. *Nature*.

[14] Kantor B., Tagliafierro L., Gu J. (2018). Downregulation of *SNCA* Expression by Targeted Editing of DNA Methylation: A Potential Strategy for Precision Therapy in PD. *Molecular Therapy*.

[15] Grobarczyk B., Franco B., Hanon K., Malgrange B. (2015). Generation of isogenic human iPS cell line precisely corrected by genome editing using the CRISPR/Cas9 system. *Stem Cell Reviews and Reports*.

[16] Polymeropoulos M. H., Lavedan C., Leroy E. (1997). Mutation in the alpha-synuclein gene identified in families with Parkinson's disease. *Science*.

[17] Deas E., Cremades N., Angelova P. R. (2016). Alpha-Synuclein oligomers interact with metal ions to induce oxidative stress and neuronal death in Parkinson's disease. *Antioxidants & Redox Signaling*.

[18] Kamal M. A., Mushtaq G., Greig N. H. (2015). Current update on synopsis of miRNA dysregulation in neurological disorders. *CNS & Neurological Disorders Drug Targets*.

[19] Singleton A. B., Farrer M., Johnson J. (2003). Alpha-synuclein locus triplication causes Parkinson's disease. *Science*.

[20] Devine M. J., Ryten M., Vodicka P. (2011). Parkinson's disease induced pluripotent stem cells with triplication of the *α*-synuclein locus. *Nature Communications*.

[21] Gründemann J., Schlaudraff F., Haeckel O., Liss B. (2008). Elevated *α*-synuclein mRNA levels in individual UV-laser-microdissected dopaminergic substantia nigra neurons in idiopathic Parkinson's disease. *Nucleic Acids Research*.

[22] Byers B., Cord B., Nguyen H. N. (2011). SNCA triplication Parkinson's patient's iPSC-derived DA neurons accumulate alpha-synuclein and are susceptible to oxidative stress. *PLoS One*.

[23] Flierl A., Oliveira L. M. A., Falomir-Lockhart L. J. (2014). Higher vulnerability and stress sensitivity of neuronal precursor cells carrying an alpha-synuclein gene triplication. *PLoS One*.

[24] Bahmad H. F., Darwish B., Dargham K. B. (2019). Role of microRNAs in anesthesia-induced neurotoxicity in animal models and neuronal cultures: a systematic review. *Neurotoxicity Research*.

[25] Neely M. D., Davison C. A., Aschner M., Bowman A. B. (2017). From the cover: manganese and rotenone-induced oxidative stress signatures differ in iPSC-derived human dopamine neurons. *Toxicological Sciences*.

[26] Heman-Ackah S. M., Manzano R., Hoozemans J. J. M. (2017). Alpha-synuclein induces the unfolded protein response in Parkinson's disease SNCA triplication iPSC-derived neurons. *Human Molecular Genetics*.

[27] Maroteaux L., Campanelli J. T., Scheller R. H. (1988). Synuclein a neuron-specific protein localized to the nucleus and presynaptic nerve terminal. *The Journal of Neuroscience*.

[28] Kontopoulos E., Parvin J. D., Feany M. B. (2006). Alpha-synuclein acts in the nucleus to inhibit histone acetylation and promote neurotoxicity. *Human Molecular Genetics*.

[29] Zhou M., Xu S., Mi J., Uéda K., Chan P. (2013). Nuclear translocation of alpha-synuclein increases susceptibility of MES23.5 cells to oxidative stress. *Brain Research*.

[30] Milanese C., Cerri S., Ulusoy A. (2018). Activation of the DNA damage response in vivo in synucleinopathy models of Parkinson's disease. *Cell Death & Disease*.

[31] Tagliafierro L., Zamora M. E., Chiba-Falek O. (2019). Multiplication of the SNCA locus exacerbates neuronal nuclear aging. *Human Molecular Genetics*.

[32] Ma K. L., Song L. K., Yuan Y. H. (2014). The nuclear accumulation of alpha-synuclein is mediated by importin alpha and promotes neurotoxicity by accelerating the cell cycle. *Neuropharmacology*.

[33] Little D., Luft C., Mosaku O. (2018). A single cell high content assay detects mitochondrial dysfunction in iPSC- derived neurons with mutations in *SNCA*. *Scientific Reports*.

[34] Zambon F., Cherubini M., Fernandes H. J. R. (2019). Cellular *α*-synuclein pathology is associated with bioenergetic dysfunction in Parkinson's iPSC-derived dopamine neurons. *Human Molecular Genetics*.

[35] Wang X., Becker K., Levine N. (2019). Pathogenic alpha-synuclein aggregates preferentially bind to mitochondria and affect cellular respiration. *Acta Neuropathologica Communications*.

[36] Ludtmann M. H. R., Angelova P. R., Horrocks M. H. (2018). *α*-synuclein oligomers interact with ATP synthase and open the permeability transition pore in Parkinson's disease. *Nature Communications*.

[37] Paillusson S., Gomez-Suaga P., Stoica R. (2017). *α*-Synuclein binds to the ER-mitochondria tethering protein VAPB to disrupt Ca^2+^ homeostasis and mitochondrial ATP production. *Acta Neuropathologica*.

[38] Ryan T., Bamm V. V., Stykel M. G. (2018). Cardiolipin exposure on the outer mitochondrial membrane modulates alpha-synuclein. *Nature Communications*.

[39] Hu D., Sun X., Liao X. (2019). Alpha-synuclein suppresses mitochondrial protease ClpP to trigger mitochondrial oxidative damage and neurotoxicity. *Acta Neuropathologica*.

[40] Wong Y. C., Holzbaur E. L. F. (2015). Autophagosome dynamics in neurodegeneration at a glance. *Journal of Cell Science*.

[41] Mazzulli J. R., Xu Y. H., Sun Y. (2011). Gaucher disease glucocerebrosidase and alpha-synuclein form a bidirectional pathogenic loop in synucleinopathies. *Cell*.

[42] Stojkovska I., Krainc D., Mazzulli J. R. (2018). Molecular mechanisms of alpha-synuclein and GBA1 in Parkinson's disease. *Cell and Tissue Research*.

[43] Mazzulli J. R., Zunke F., Isacson O., Studer L., Krainc D. (2016). Alpha-synuclein-induced lysosomal dysfunction occurs through disruptions in protein trafficking in human midbrain synucleinopathy models. *Proceedings of the National Academy of Sciences of the United States of America*.

[44] Mazzulli J. R., Zunke F., Tsunemi T. (2016). Activation of beta-glucocerebrosidase reduces pathological alpha-synuclein and restores lysosomal function in Parkinson's patient midbrain neurons. *The Journal of Neuroscience*.

[45] Haenseler W., Zambon F., Lee H. (2017). Excess *α*-synuclein compromises phagocytosis in iPSC-derived macrophages. *Scientific Reports*.

[46] Goldstein A. Y. N., Wang X., Schwarz T. L. (2008). Axonal transport and the delivery of pre-synaptic components. *Current Opinion in Neurobiology*.

[47] Garcia-Reitböck P., Anichtchik O., Bellucci A. (2010). SNARE protein redistribution and synaptic failure in a transgenic mouse model of Parkinson's disease. *Brain*.

[48] Janezic S., Threlfell S., Dodson P. D. (2013). Deficits in dopaminergic transmission precede neuron loss and dysfunction in a new Parkinson model. *Proceedings of the National Academy of Sciences of the United States of America*.

[49] Schirinzi T., Madeo G., Martella G. (2016). Early synaptic dysfunction in Parkinson's disease: insights from animal models. *Movement Disorders*.

[50] Prots I., Veber V., Brey S. (2013). *α*-Synuclein oligomers impair neuronal microtubule-kinesin interplay. *The Journal of Biological Chemistry*.

[51] Oikawa T., Nonaka T., Terada M., Tamaoka A., Hisanaga S. I., Hasegawa M. (2016). *α*-Synuclein fibrils exhibit gain of toxic function, promoting tau aggregation and inhibiting microtubule assembly. *The Journal of Biological Chemistry*.

[52] Li X., James S., Lei P. (2016). Interactions between *α*-Synuclein and tau protein: implications to neurodegenerative disorders. *Journal of Molecular Neuroscience*.

[53] Prots I., Grosch J., Brazdis R. M. (2018). *α*-Synuclein oligomers induce early axonal dysfunction in human iPSC-based models of synucleinopathies. *Proceedings of the National Academy of Sciences of the United States of America*.

[54] Martin I., Kim J. W., Dawson V. L., Dawson T. M. (2014). LRRK2 pathobiology in Parkinson's disease. *Journal of Neurochemistry*.

[55] Liu G. H., Qu J., Suzuki K. (2012). Progressive degeneration of human neural stem cells caused by pathogenic LRRK2. *Nature*.

[56] Cheng Y. C., Huang C. Y., Ho M. C. (2018). Generation of 2 induced pluripotent stem cell lines derived from patients with Parkinson's disease carrying LRRK2 G2385R variant. *Stem Cell Research*.

[57] Ma D., Zhou W., Ng E. Y., Zeng L., Zhao Y., Tan E. K. (2017). Reprogramming of a human induced pluripotent stem cell (iPSC) line from a Parkinson's disease patient with a R1628P variant in the LRRK2 gene. *Stem Cell Research*.

[58] Ma D., Ng E. Y., Zeng L., Lim C. Y. Y., Zhao Y., Tan E. K. (2017). Development of a human induced pluripotent stem cell (iPSC) line from a Parkinson's disease patient carrying the N551K variant in LRRK2 gene. *Stem Cell Research*.

[59] Ma D., Ng S. H., Zeng L., Zhao Y., Tan E. K. (2017). Generation of a human induced pluripotent stem cell (iPSC) line carrying the Parkinson's disease linked LRRK2 variant S1647T. *Stem Cell Research*.

[60] Nguyen H. N., Byers B., Cord B. (2011). LRRK2 mutant iPSC-derived DA neurons demonstrate increased susceptibility to oxidative stress. *Cell Stem Cell*.

[61] Bieri G., Brahic M., Bousset L. (2019). LRRK2 modifies *α*-syn pathology and spread in mouse models and human neurons. *Acta Neuropathologica*.

[62] Daher J. P. (2017). Interaction of LRRK2 and *α*-synuclein in Parkinson's disease. *Advances in Neurobiology*.

[63] Bahnassawy L.'a., Nicklas S., Palm T. (2013). The Parkinson's disease-associated LRRK2 mutation R1441G inhibits neuronal differentiation of neural stem cells. *Stem Cells and Development*.

[64] Borgs L., Peyre E., Alix P. (2016). Dopaminergic neurons differentiating from *LRRK2* G2019S induced pluripotent stem cells show early neuritic branching defects. *Scientific Reports*.

[65] Schwab A. J., Ebert A. D. (2015). Neurite aggregation and calcium dysfunction in iPSC-derived sensory neurons with Parkinson's disease-related LRRK2 G2019S mutation. *Stem Cell Reports*.

[66] Korecka J. A., Talbot S., Osborn T. M. (2019). Neurite collapse and altered ER Ca^2+^ control in human Parkinson disease patient iPSC-derived neurons with LRRK2 G2019S mutation. *Stem Cell Reports*.

[67] Walter J., Bolognin S., Antony P. M. A. (2019). Neural stem cells of Parkinson's disease patients exhibit aberrant mitochondrial morphology and functionality. *Stem Cell Reports*.

[68] Sanders L. H., Laganière J., Cooper O. (2014). *LRRK2* mutations cause mitochondrial DNA damage in iPSC-derived neural cells from Parkinson's disease patients: Reversal by gene correction. *Neurobiology of Disease*.

[69] Howlett E. H., Jensen N., Belmonte F. (2017). LRRK2 G2019S-induced mitochondrial DNA damage is LRRK2 kinase dependent and inhibition restores mtDNA integrity in Parkinson’s disease. *Human Molecular Genetics*.

[70] Hsieh C. H., Shaltouki A., Gonzalez A. E. (2016). Functional impairment in Miro degradation and mitophagy is a shared feature in familial and sporadic Parkinson's disease. *Cell Stem Cell*.

[71] Schwab A. J., Sison S. L., Meade M. R., Broniowska K. A., Corbett J. A., Ebert A. D. (2017). Decreased sirtuin deacetylase activity in LRRK2 G2019S iPSC-derived dopaminergic neurons. *Stem Cell Reports*.

[72] Pan P. Y., Li X., Wang J. (2017). Parkinson's disease-associated LRRK2 hyperactive kinase mutant disrupts synaptic vesicle trafficking in ventral midbrain neurons. *The Journal of Neuroscience*.

[73] Connor-Robson N., Booth H., Martin J. G. (2019). An integrated transcriptomics and proteomics analysis reveals functional endocytic dysregulation caused by mutations in LRRK2. *Neurobiology of Disease*.

[74] Nguyen M., Krainc D. (2018). LRRK2 phosphorylation of auxilin mediates synaptic defects in dopaminergic neurons from patients with Parkinson's disease. *Proceedings of the National Academy of Sciences of the United States of America*.

[75] Schapansky J., Khasnavis S., DeAndrade M. (2018). Familial knockin mutation of LRRK2 causes lysosomal dysfunction and accumulation of endogenous insoluble *α*-synuclein in neurons. *Neurobiology of Disease*.

[76] Su Y. C., Qi X. (2013). Inhibition of excessive mitochondrial fission reduced aberrant autophagy and neuronal damage caused by LRRK2 G2019S mutation. *Human Molecular Genetics*.

[77] Ho D. H., Kim H., Nam D. (2018). LRRK2 impairs autophagy by mediating phosphorylation of leucyl-tRNA synthetase. *Cell Biochemistry and Function*.

[78] Lee H., James W. S., Cowley S. A. (2017). LRRK2 in peripheral and central nervous system innate immunity: its link to Parkinson's disease. *Biochemical Society Transactions*.

[79] López de Maturana R., Lang V., Zubiarrain A. (2016). Mutations in LRRK2 impair NF-*κ*B pathway in iPSC-derived neurons. *Journal of Neuroinflammation*.

[80] Booth H. D. E., Wessely F., Connor-Robson N. (2019). RNA sequencing reveals MMP2 and TGFB1 downregulation in *LRRK2* G2019S Parkinson's iPSC-derived astrocytes. *Neurobiology of Disease*.

[81] Speidel A., Felk S., Reinhardt P., Sterneckert J., Gillardon F. (2016). Leucine-rich repeat kinase 2 influences fate decision of human monocytes differentiated from induced pluripotent stem cells. *PLoS One*.

[82] Wood-Kaczmar A., Gandhi S., Yao Z. (2008). PINK1 is necessary for long term survival and mitochondrial function in human dopaminergic neurons. *PLoS One*.

[83] Imaizumi Y., Okada Y., Akamatsu W. (2012). Mitochondrial dysfunction associated with increased oxidative stress and *α*-synuclein accumulation in PARK2 iPSC-derived neurons and postmortem brain tissue. *Molecular Brain*.

[84] Tabata Y., Imaizumi Y., Sugawara M. (2018). T-type calcium channels determine the vulnerability of dopaminergic neurons to mitochondrial stress in familial Parkinson disease. *Stem Cell Reports*.

[85] Chung S. Y., Kishinevsky S., Mazzulli J. R. (2016). Parkin and PINK1 Patient iPSC-Derived Midbrain Dopamine Neurons Exhibit Mitochondrial Dysfunction and *α*-Synuclein Accumulation. *Stem Cell Reports*.

[86] Cooper O., Seo H., Andrabi S. (2012). Pharmacological rescue of mitochondrial deficits in iPSC-derived neural cells from patients with familial Parkinson's disease. *Science Translational Medicine*.

[87] Seibler P., Graziotto J., Jeong H., Simunovic F., Klein C., Krainc D. (2011). Mitochondrial Parkin recruitment is impaired in neurons derived from mutant PINK1 induced pluripotent stem cells. *The Journal of Neuroscience*.

[88] Vos M., Geens A., Böhm C. (2017). Cardiolipin promotes electron transport between ubiquinone and complex I to rescue PINK1 deficiency. *The Journal of Cell Biology*.

[89] Shaltouki A., Sivapatham R., Pei Y. (2015). Mitochondrial Alterations by PARKIN in Dopaminergic Neurons Using PARK2 Patient-Specific and *PARK2* Knockout Isogenic iPSC Lines. *Stem Cell Reports*.

[90] Zanon A., Kalvakuri S., Rakovic A. (2017). SLP-2 interacts with Parkin in mitochondria and prevents mitochondrial dysfunction in Parkin-deficient human iPSC-derived neurons and *Drosophila*. *Human Molecular Genetics*.

[91] Zanon A., Kalvakuri S., Rakovic A. (2019). Corrigendum: SLP-2 interacts with Parkin in mitochondria and prevents mitochondrial dysfunction in Parkin-deficient human iPSC-derived neurons and *Drosophila*. *Human Molecular Genetics*.

[92] Pacelli C., Rotundo G., Lecce L. (2019). Parkin mutation affects clock gene-dependent energy metabolism. *International Journal of Molecular Sciences*.

[93] Nguyen T. N., Padman B. S., Lazarou M. (2016). Deciphering the molecular signals of PINK1/Parkin mitophagy. *Trends in Cell Biology*.

[94] Rakovic A., Shurkewitsch K., Seibler P. (2013). Phosphatase and tensin homolog (PTEN)-induced putative kinase 1 (PINK1)-dependent ubiquitination of endogenous Parkin attenuates mitophagy: study in human primary fibroblasts and induced pluripotent stem cell-derived neurons. *The Journal of Biological Chemistry*.

[95] Narendra D. P., Jin S. M., Tanaka A. (2010). PINK1 is selectively stabilized on impaired mitochondria to activate Parkin. *PLoS Biology*.

[96] Oh C.-K., Sultan A., Platzer J. (2017). S-Nitrosylation of PINK1 attenuates PINK1/Parkin-dependent mitophagy in hiPSC-based Parkinson’s disease models. *Cell Reports*.

[97] Suzuki S., Akamatsu W., Kisa F. (2017). Efficient induction of dopaminergic neuron differentiation from induced pluripotent stem cells reveals impaired mitophagy in PARK2 neurons. *Biochemical and Biophysical Research Communications*.

[98] Jiang H., Ren Y., Yuen E. Y. (2012). Parkin controls dopamine utilization in human midbrain dopaminergic neurons derived from induced pluripotent stem cells. *Nature Communications*.

[99] Zhong P., Hu Z., Jiang H., Yan Z., Feng J. (2017). Dopamine induces oscillatory activities in human midbrain neurons with Parkin mutations. *Cell Reports*.

[100] Ren Y., Zhao J., Feng J. (2003). Parkin binds to alphabeta tubulin and increases their ubiquitination and degradation. *The Journal of Neuroscience*.

[101] Yang F., Jiang Q., Zhao J., Ren Y., Sutton M. D., Feng J. (2005). Parkin stabilizes microtubules through strong binding mediated by three independent domains. *The Journal of Biological Chemistry*.

[102] Ren Y., Jiang H., Yang F., Nakaso K., Feng J. (2009). Parkin protects dopaminergic neurons against microtubule-depolymerizing toxins by attenuating microtubule-associated protein kinase activation. *The Journal of Biological Chemistry*.

[103] Ren Y., Jiang H., Hu Z. (2015). Parkin mutations reduce the complexity of neuronal processes in iPSC-derived human neurons. *Stem Cells*.

[104] Cartelli D., Amadeo A., Calogero A. M. (2018). Parkin absence accelerates microtubule aging in dopaminergic neurons. *Neurobiology of Aging*.

[105] Nguyen M., Wong Y. C., Ysselstein D., Severino A., Krainc D. (2019). Synaptic, mitochondrial, and lysosomal dysfunction in Parkinson's disease. *Trends in Neurosciences*.

[106] Vilariño-Güell C., Wider C., Ross O. A. (2011). *VPS35* Mutations in Parkinson Disease. *American Journal of Human Genetics*.

[107] Zimprich A., Benet-Pagès A., Struhal W. (2011). A Mutation in *VPS35*, Encoding a Subunit of the Retromer Complex, Causes Late-Onset Parkinson Disease. *American Journal of Human Genetics*.

[108] Munsie L. N., Milnerwood A. J., Seibler P. (2015). Retromer-dependent neurotransmitter receptor trafficking to synapses is altered by the Parkinson's disease VPS35 mutation p.D620N. *Human Molecular Genetics*.

[109] Sidransky E., Nalls M. A., Aasly J. O. (2009). Multicenter analysis of glucocerebrosidase mutations in Parkinson's disease. *The New England Journal of Medicine*.

[110] Woodard C. M., Campos B. A., Kuo S. H. (2014). iPSC-derived dopamine neurons reveal differences between monozygotic twins discordant for Parkinson's disease. *Cell Reports*.

[111] Aflaki E., Borger D. K., Moaven N. (2016). A new glucocerebrosidase chaperone Reduces *α*-Synuclein and glycolipid levels in iPSC-derived dopaminergic neurons from patients with Gaucher disease and parkinsonism. *The Journal of Neuroscience*.

[112] Kim S., Yun S. P., Lee S. (2018). GBA1 deficiency negatively affects physiological *α*-synuclein tetramers and related multimers. *Proceedings of the National Academy of Sciences of the United States of America*.

[113] Taguchi Y. V., Liu J., Ruan J. (2017). Glucosylsphingosine promotes *α*-Synuclein pathology in mutant GBA-associated Parkinson's disease. *The Journal of Neuroscience*.

[114] Barkhuizen M., Anderson D. G., Grobler A. F. (2016). Advances in GBA-associated Parkinson's disease - Pathology, presentation and therapies. *Neurochemistry International*.

[115] Fernandes H. J. R., Hartfield E. M., Christian H. C. (2016). ER Stress and Autophagic Perturbations Lead to Elevated Extracellular *α*-Synuclein in *GBA-N370S* Parkinson's iPSC-Derived Dopamine Neurons. *Stem Cell Reports*.

[116] Schöndorf D. C., Ivanyuk D., Baden P. (2018). The NAD+ precursor nicotinamide riboside rescues mitochondrial defects and neuronal loss in iPSC and fly models of Parkinson's disease. *Cell Reports*.

[117] Brundin P., Melki R. (2017). Prying into the prion hypothesis for Parkinson's disease. *The Journal of Neuroscience*.

[118] Holmes B. B., DeVos S. L., Kfoury N. (2013). Heparan sulfate proteoglycans mediate internalization and propagation of specific proteopathic seeds. *Proceedings of the National Academy of Sciences of the United States of America*.

[119] Freundt E. C., Maynard N., Clancy E. K. (2012). Neuron-to-neuron transmission of *α*-synuclein fibrils through axonal transport. *Annals of Neurology*.

[120] Yamasaki T. R., Holmes B. B., Furman J. L. (2019). Parkinson's disease and multiple system atrophy have distinct *α*-synuclein seed characteristics. *The Journal of Biological Chemistry*.

[121] Bengoa-Vergniory N., Roberts R. F., Wade-Martins R., Alegre-Abarrategui J. (2017). Alpha-synuclein oligomers: a new hope. *Acta Neuropathologica*.

[122] Gribaudo S., Tixador P., Bousset L. (2019). Propagation of *α*-Synuclein Strains within Human Reconstructed Neuronal Network. *Stem Cell Reports*.

[123] Surguchev A. A., Emamzadeh F. N., Surguchov A. (2019). Cell responses to extracellular *α*-Synuclein. *Molecules*.

[124] Okita K., Nakagawa M., Hyenjong H., Ichisaka T., Yamanaka S. (2008). Generation of mouse induced pluripotent stem cells without viral vectors. *Science*.

[125] Lowry W. E., Plath K. (2008). The many ways to make an iPS cell. *Nature Biotechnology*.

[126] Zhou Y. Y., Zeng F. (2013). Integration-free methods for generating induced pluripotent stem cells. *Genomics, Proteomics & Bioinformatics*.

[127] Warren L., Manos P. D., Ahfeldt T. (2010). Highly efficient reprogramming to pluripotency and directed differentiation of human cells with synthetic modified mRNA. *Cell Stem Cell*.

[128] Mandal P. K., Rossi D. J. (2013). Reprogramming human fibroblasts to pluripotency using modified mRNA. *Nature Protocols*.

[129] Kogut I., McCarthy S. M., Pavlova M. (2018). High-efficiency RNA-based reprogramming of human primary fibroblasts. *Nature Communications*.

[130] Zhou H., Wu S., Joo J. Y. (2009). Generation of induced pluripotent stem cells using recombinant proteins. *Cell Stem Cell*.

[131] Chen F., Zhang G., Yu L. (2016). High-efficiency generation of induced pluripotent mesenchymal stem cells from human dermal fibroblasts using recombinant proteins. *Stem Cell Research & Therapy*.

[132] Huangfu D., Osafune K., Maehr R. (2008). Induction of pluripotent stem cells from primary human fibroblasts with only *Oct4* and *Sox2*. *Nature Biotechnology*.

[133] Zhu S., Li W., Zhou H. (2010). Reprogramming of human primary somatic cells by OCT4 and chemical compounds. *Cell Stem Cell*.

[134] Biswas D., Jiang P. (2016). Chemically induced reprogramming of somatic cells to pluripotent stem cells and neural cells. *International Journal of Molecular Sciences*.

[135] Alaaeddine R., Fayad M., Nehme E., Bahmad H. F., Kobeissy F. (2017). The emerging role of proteomics in precision medicine: applications in neurodegenerative diseases and neurotrauma. *Advances in Experimental Medicine and Biology*.

[136] Yamashita A., Morioka M., Kishi H. (2014). Statin treatment rescues FGFR3 skeletal dysplasia phenotypes. *Nature*.

[137] Awad O., Sarkar C., Panicker L. M. (2015). Altered TFEB-mediated lysosomal biogenesis in Gaucher disease iPSC-derived neuronal cells. *Human Molecular Genetics*.

[138] Chang E. A., Tomov M. L., Suhr S. T. (2015). Derivation of ethnically diverse human induced pluripotent stem cell lines. *Scientific Reports*.

[139] Barker R. A., TRANSEURO consortium (2019). Designing stem-cell-based dopamine cell replacement trials for Parkinson's disease. *Nature Medicine*.

[140] Olanow C. W., Freeman T., Kordower J. (2001). Transplantation of embryonic dopamine neurons for severe Parkinson's disease. *The New England Journal of Medicine*.

[141] Allan L. E., Petit G. H., Brundin P. (2010). Cell transplantation in Parkinson's disease: problems and perspectives. *Current Opinion in Neurology*.

[142] de Lazaro I., Yilmazer A., Kostarelos K. (2014). Induced pluripotent stem (iPS) cells: a new source for cell-based therapeutics?. *Journal of Controlled Release*.

[143] Doi D., Samata B., Katsukawa M. (2014). Isolation of human induced pluripotent stem cell-derived dopaminergic progenitors by cell sorting for successful transplantation. *Stem Cell Reports*.

[144] Samata B., Doi D., Nishimura K. (2016). Purification of functional human ES and iPSC-derived midbrain dopaminergic progenitors using LRTM1. *Nature Communications*.

[145] Bye C. R., Jönsson M. E., Björklund A., Parish C. L., Thompson L. H. (2015). Transcriptome analysis reveals transmembrane targets on transplantable midbrain dopamine progenitors. *Proceedings of the National Academy of Sciences of the United States of America*.

[146] Hallett P. J., Deleidi M., Astradsson A. (2015). Successful function of autologous iPSC-derived dopamine neurons following transplantation in a non-human primate model of Parkinson's disease. *Cell Stem Cell*.

[147] Song B., Cha Y., Ko S. (2020). Human autologous iPSC–derived dopaminergic progenitors restore motor function in Parkinson's disease models. *The Journal of Clinical Investigation*.

[148] Xiang M., Lu M., Quan J. (2019). Directin vivoapplication of induced pluripotent stem cells is feasible and can be safe. *Theranostics*.

[149] Takahashi J. (2017). Strategies for bringing stem cell-derived dopamine neurons to the clinic: the Kyoto trial. *Progress in Brain Research*.

[150] Takahashi J. (2019). Preparing for first human trial of induced pluripotent stem cell-derived cells for Parkinson's disease: an interview with Jun Takahashi. *Regenerative Medicine*.

[151] Morizane A. (2019). Cell therapy for Parkinson's disease with induced pluripotent stem cells. *Rinshō Shinkeigaku*.

[152] Qian X., Nguyen H. N., Song M. M. (2016). Brain-region-specific organoids using mini-bioreactors for modeling ZIKV exposure. *Cell*.

[153] Lancaster M. A., Renner M., Martin C. A. (2013). Cerebral organoids model human brain development and microcephaly. *Nature*.

[154] Paşca A. M., Sloan S. A., Clarke L. E. (2015). Functional cortical neurons and astrocytes from human pluripotent stem cells in 3D culture. *Nature Methods*.

[155] Lee C. T., Chen J., Kindberg A. A. (2017). CYP3A5 Mediates Effects of Cocaine on Human Neocorticogenesis: Studies using an *In Vitro* 3D Self-Organized hPSC Model with a Single Cortex-Like Unit. *Neuropsychopharmacology*.

[156] Suhr S. T., Chang E. A., Rodriguez R. M. (2009). Telomere dynamics in human cells reprogrammed to pluripotency. *PLoS One*.

[157] Yehezkel S., Rebibo-Sabbah A., Segev Y. (2011). Reprogramming of telomeric regions during the generation of human induced pluripotent stem cells and subsequent differentiation into fibroblast-like derivatives. *Epigenetics*.

[158] Rohani L., Johnson A. A., Arnold A., Stolzing A. (2014). The aging signature a hallmark of induced pluripotent stem cells. *Aging Cell*.

[159] Miller J. D., Ganat Y. M., Kishinevsky S. (2013). Human iPSC-based modeling of late-onset disease via progerin-induced aging. *Cell Stem Cell*.

[160] Gunhanlar N., Shpak G., van der Kroeg M. (2018). A simplified protocol for differentiation of electrophysiologically mature neuronal networks from human induced pluripotent stem cells. *Molecular Psychiatry*.

